# A comparative study of stress experienced by Swedish and Norwegian police officers

**DOI:** 10.3389/frhs.2023.1072248

**Published:** 2023-02-27

**Authors:** Mojgan Padyab, Jonas Hansson, Johanna Sundqvist, Miguel Inzunza, Mehdi Ghazinour

**Affiliations:** ^1^ Centre for Demographic and Ageing Research, Faculty of Social Sciences, Umeå University, Umeå, Sweden; ^2^ Police Education Unit, Faculty of Social Sciences, Umeå University, Umeå, Sweden; ^3^ Department of Social Work, Umeå University, Umeå, Sweden

**Keywords:** stress, police, comparative study, Sweden, Norway

## Abstract

**Introduction:**

Police officers work in a variable environment under different circumstances and often involves stressful situations. This include working irregular hours, ongoing exposure to critical incidents, confrontations and violence. community police officers are mainly out in the society and have daily contact with the general public. critical incidents can also consist of being criticized and stigmatized as a police officer, both from the public but also lack of support from their own organization. There is evidence on negative impacts of stress on police officers. However, knowledge about the nature of police stress and its various types is insufficient. It is assumed that there are common stress factors which are universal among all police officers in different contexts but there is a lack of comparative studies to provide empirical evidence. The aim of this study is to compare different types of stress among police officers in Norway and Sweden and how the pattern of experiencing stress has changed over time in these countries.

**Methods:**

The study population consisted of patrolling police officers from 20 local police districts or units in all seven regions in Sweden (*n* = 953) and patrolling police officers from four police districts in Norway (*n* = 678). A 42-item Police Stress Identification Questionnaire was used to measure the stress level.

**Results:**

The findings show differences in types of stressful events as well as its severity among police officers in Sweden and Norway. The level of stress decreased over time among Swedish police officers whereas it showed no change or even an increase among the Norwegian participants.

**Discussion:**

The results of this study are relevant for policy-makers, police authorities and lay police officers in each country to tailor their efforts to prevent stress among police officers.

## 1. Introduction

Police officers are exposed to emotionally demanding situations, which come with the job. These situations include not only being confronted with death and illness and dealing with crime and accident victims but also interacting with perpetrators and disturbing persons and being exposed to various types of threats (1, 2). Police work includes regular and ongoing exposure to confrontation, violence, and, at its most extreme, potential harm to the officer's life (3). Many of these situations involve vulnerable people who are expected to receive support from the police (4). Therefore, police officers are continuously exposed to critical incidents as a part of their daily work. However, these critical incidents can also involve being criticized and stigmatized by the public and receiving little support from their own organization (5). For example, in a study on citizens' decisions to complain about the police, McLean (6) showed that the likelihood of complaining increases when the citizen perceives the interaction as procedurally and distributively unfair and when the outcome is unfavorable. In addition, police officers often work irregular hours, a factor that has been seen to negatively affect their health (e.g., an increased risk of cardiovascular disease) (7).

In summary, the complexity of demanding work-related situations and the factors described above have been shown to affect the stress levels of police officers (8–10). Although many investigations in police research have indicated the role of stress in police work, little is known about the various types of police stress. Without a comparative approach, it is difficult to determine whether there are some types of stress that are common among all police officers. In this comparative study, we aim to contribute to police stress research, identify specific types of police stress in Sweden and Norway, and assess the severity of these stress factors in both countries. We argue that this issue is highly relevant to police work and the profession because the identification of specific stress factors in police work creates the opportunity to develop resilience programs aimed at preventing long-term negative psychiatric, psychological, and social consequences for police officers. Moreover, the identification of police stress contributes to self-awareness among police officers and to police education by supporting the development of theoretical and practical modules to prepare police trainees for future police work. Researchers have reported the long-term effects of stress on police officers, which include maladaptive and antisocial behaviors, such as problem drinking, hyper-aggressiveness, and violence in work and private life (11, 12).

The current study compares Norway and Sweden because of the many similarities in their criminal justice systems (13); for example, the police are unified and state-organized, and each country is divided into police districts with a considerable degree of independence (13). However, there also exist several differences, including those in the way they organize prosecution, the number of police per resident, and the regulations concerning force resources (e.g., pepper spray and firearms). Furthermore, police work is carried out differently in Sweden and Norway. Norwegian police do not carry firearms unless the situation is expected to merit it (14), this is derived from that the police adopt a philosophy of policing by consent rather than policing with the threat of force.

## 2. Theoretical framework

Stress is defined as human psychological and physiological adaptation to the environment (15). Coping is defined as a cognitive resource for managing stressful internal and external situations (16). The coping theory introduced by Folkman and Lazarus contributes to increasing our understanding of the relationship between human cognition and the environment through primary and secondary appraisals. In their early studies on coping, Folkman and Lazarus suggested two coping mechanisms: problem-focused and emotion-focused coping. Later, two other main mechanisms of coping, namely, meaning-focused and support-seeking coping, were presented (16). Adding John Violanti's research on police stress from the early 1990 s until now and the long-term consequences of exposure to massive stress creates an opportunity to comprehensively study the stressful work environment of police (16–22). In this article we use coping theory as our point of departure. Police work requires mental preparation before acting or managing complex threatful situations. Given that Coping theory is based on individual's appraisal, it would contribute in understanding how police officers recognize the source of stress and how they organize their policing based on their judgment of the stressful event. Stress in police work.

Many studies support the notion that police work is highly stressful (9, 23–26) and that police officers experience high levels of work-related stress relative to many other occupations (27). For example, working with traumatized victims and conducting homicide inquiries are stressors in the area of criminal investigation (28–30). Police work also involves shift work, traumatic events, staff shortages, and bureaucratic burden, all of which are considered stressful. Lucas and colleagues (31) found that killing in the line of duty, inadequate supervisor support, departmental politics, and insufficient personnel are work stressors that are rated as especially stressful; meanwhile, assignment to nonpolicing duties, demand for high morality, promotion, and boredom are not rated as especially stressful. Job dissatisfaction, workplace discrimination, lack of cooperation among coworkers, and exposure to critical incidents are associated with perceived work stress (32). Additionally, studies have addressed different stress patterns among female and male police officers (33, 34). Some studies have explored the relationship between police officers' strategies to cope with stress and police culture (10) while other studies have focused on individual factors, such as age and gender, in relation to police officers' coping strategies (35). Gershon and colleagues (32) found that avoidant or negative coping is associated with higher levels of perceived work stress and adverse health outcomes.

The present study focused on the comparison of stress levels between police officers in Sweden and Norway and the temporal changes over time.

## 3. Methods

### 3.1. Setting

This study comprises police officers from two neighboring Scandinavian countries, namely, Sweden and Norway.

#### 3.1.1. The Swedish police

The Swedish police is a national authority with seven geographical police regions comprising 100 local police districts. In 2018, approximately 30% of 20,040 police officers were women, and the average age of all officers was 44 years. The Swedish Police Authority is led by the National Police Commissioner.

#### 3.1.2. The Norwegian police

The Norwegian police is a national authority consisting of 12 police districts and is mainly responsible for police operations within a geographical area. There are approximately 10,000 police officers, approximately 30% of whom are women.

### 3.2. Data collection

Data originated from three investigations: the Mareld study (36) in Sweden and two studies aimed at evaluating conducted energy weapons (CEWs) in Sweden and Norway. A Conducted Energy Weapon (CEW) is a device that delivers low amperage electrical current into its target, temporarily impacting the sensory and motor nervous system. When properly deployed, a CEW can disable an assailant with limited risk of long term or substantial injury. Response rate was 52% in the Swedish sample and 56% in the Norwegian sample. The authors were assigned by the Stockholm Police Region to conduct a scientific investigation (Mareld) with a focus on the health and working conditions among police officers working in so-called vulnerable areas in Stockholm, a term applied by the Swedish Police to areas with high crime rates and social exclusion. The data were collected from 2017 to 2020. In addition, the authors evaluated the test periods on CEWs of the Swedish and Norwegian police forces. The Swedish test period was between 2018 and 2019, and the Norwegian test period was between 2019 and 2020. In all three investigations, data were collected annually in three phases.

All police officers present at work were eligible to take part in the study. Participation was voluntary and no incentive was given to participants. The entire sample consisted of patrolling police officers from 20 local police districts or units in all seven regions of Sweden (*n* = 954) and patrolling police officers from 4 police districts in Norway (*n* = 677).

#### 3.2.1. Sample and data collection in the Mareld study

The researchers visited three local police districts in the Stockholm Police Region in spring 2018 and spring 2019 and personally informed them about the project and ethical issues to obtain informed consent. The project was described in a letter and a paper survey was distributed. The third data collection in spring 2020 was modified because of the COVID-19 pandemic; specifically, one appointed police officer in every three local police districts distributed and collected the paper survey and then sent it to us by mail. The project was described in an information letter provided to the respondents.

This study was approved by the Regional Ethical Review Board of Umeå University (Dnr 2017/516-31). This project was funded by the Stockholm Police Region.

#### 3.2.2. Sample and data collection in the Swedish CEW study

The surveys and prepaid return envelopes were sent in a sealed envelope to the contact persons at each local police district or unit for distribution to police officers who could respond to the survey and return it using the prepaid return envelopes in December 2017. The project was presented to the researchers' contacts in the police authority through written and verbal descriptions. A reminder was conveyed to the contact persons through e-mail and communicated further to the police officers. This convenience sample was used because of confidentiality rules in the Swedish police organization. In November 2018 and November 2019, a link to the web survey and an information letter were distributed through e-mail to the police officers involved in the study by the contact person within the police authority. Two reminders were sent to police officers by e-mail.

This study was approved by the Swedish Ethical Review Authority (Dnr 2019-02464). This project was funded by the Swedish Police Authority.

#### 3.2.3. Sample and data collection in the Norwegian CEW study

The dataset was based on a survey conducted among all police officers who participated in the CEW evaluation. The survey and an information letter describing the project were distributed by the National Police Directorate through e-mail and made available to all participating officers in the evaluation of CEWs. Two reminders were sent to the police officers by e-mail. Once the data were collected, they were handed over to the research team after being anonymized.

#### 3.2.4. Instrument

A 42-item Police Stress Identification Questionnaire (11) was used to measure stress levels with the following instruction: “These questions map different types of stressors that affect you as a police officer or civil servant. Mark with a cross how strong you experienced the stress. It can be both that you experienced the event or that you experienced the risk of it happening as stressful.” A numerical rating from 0 to 9 was applied, with 9 indicating the most stressful level. A previous study has reported the validity and reliability of the instrument (11). Reliability of the questionnaire was checked with internal consistency assessment methods. Consistency of the entire scale was assessed using the Cronbach's alpha coefficient which was 0.95.

To ascertain face validity, we interviewed nine police officers focusing on items provided to them and discussed them, given their personal experiences that may lead to generating new items (11). The interviewees, aged 30–60 years, had on average more than 10 years of experience in the field. They were informed about the study's aim. Each interview took approximately 30 min. The content of interviews was discussed in our authorship group using content analysis and our theoretical perspectives. Phrases identified from the content analysis were discussed in the expert panel, including one expert in the field of stress and coping and an experienced police officer, and provided the basis for modifying or rewording the items and generating new items that were not included in the preliminary list. Interview data were used to confirm concepts in the existing literature about police stress and explore new areas. The results from confirmatory factor analysis showed the highest fit for data, suggesting construct validity of the PSIQ (11).

#### 3.2.5. Statistical methods

A random-effects ordered logistic model was adopted to analyze the data. Ordered logistic models were used to compare stress levels between police officers in Sweden and Norway as well as the temporal changes over the study period. We assessed (1) whether there is a difference between Sweden and Norway; (2) any changes in the mean score of stress items over time (three time points); and (3) the interactions between country and time point, that is, whether or not the change over time differs between Sweden and Norway. This method is designed to estimate models in the presence of a variable number of waves for each individual (37). Some individuals participated once, twice, and all three times. In this structure, repeated observations (panels) are nested within individual observations. We chose an optimal statistical method that accounts for the correlation between within-person measures repeated over time. We also estimated the predicted means in the form of average marginal effects (38) of the stress items at each measurement point for each country.

All estimations were performed using Stata version 16.1 (StataCorp, College Station, TX).

## 4. Results

A total of 1,631 police officers (677 from Norway and 954 from Sweden) participated in this study (73% of them were male). Their baseline ages ranged from 22 to 66 years (mean age = 35 years; SD = 8 years).

**Figure 1:**
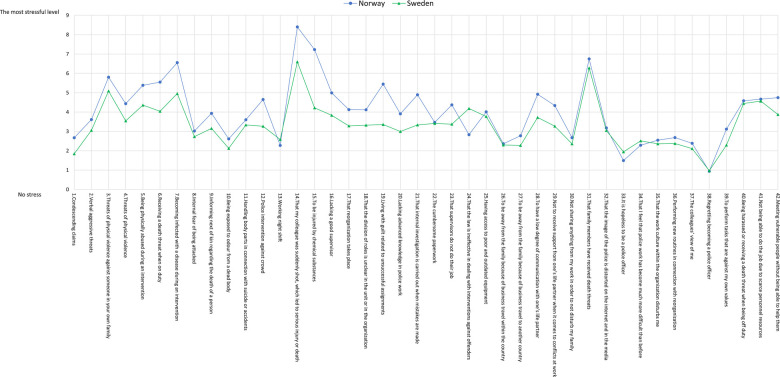
Mean score for stress items among Swedish and Norwegian police officers.

Figure 1 shows the mean scores for the stress items among the Swedish and Norwegian police officers. The police officers in both countries identified “That my colleague was suddenly shot, which led to serious injury or death” as the most stressful factor, followed by “That family members have received death threats.” Both countries identified “Regretting becoming a police officer” as the least stressful factor.

The mean score for the majority of the items (26 out of 42 items) was significantly higher among Norwegian police officers, suggesting that they experience a higher level of stress than their Swedish counterparts. Only five items were scored higher among Swedish police officers. These items are as follows:

P13. Working night shift
P24. That the law is ineffective in dealing with interventions against offenders
P33. It is hopeless to be a police officer
P34. That I feel that police work has become much more difficult than before
P38. Regretting becoming a police officer

Table 1 presents the mean scores of the 42 stress items among police officers in Sweden and Norway.

**Table 1:** Mean scores for the stress items among police officers in Sweden and Norway at three measurement points.

		Norway			Sweden			*P*-value	*P*-value	*P*-value
								Comparison over time	Comparison between countries	Interaction between time and country
Item		T1	T2	T3	T1	T2	T3			
1	Condescending claims	2.8	2.7	2.5	1.8	2.0	1.8	NS	<0.001	NS
2	Verbal aggressive threats	3.6	3.5	3.7	3.1	3.1	3.0	NS	<0.001	NS
3	Threats of physical violence against someone in your own family	5.7	5.8	6.0	5.1	5.3	5.0	NS	<0.001	NS
4	Threats of physical violence	4.3	4.4	4.6	3.6	3.5	3.5	NS	<0.001	NS
5	Being physically abused during an intervention	5.4	5.3	5.4	4.5	4.4	4.1	NS	<0.001	NS
6	Receiving a death threat when on duty	5.5	5.4	5.7	4.2	4.0	3.8	NS	<0.001	<0.002
7	Becoming infected with a disease during an intervention	6.7	6.6	6.4	5.3	4.9	4.7	<0.003	<0.001	NS
8	Internal fear of being attacked	3.1	3.0	3.0	2.9	2.7	2.6	NS	NS	NS
9	Informing next of kin regarding the death of a person	4.0	3.8	3.9	3.2	3.1	3.1	NS	<0.001	NS
10	Being exposed to odor from a dead body	2.6	2.6	2.7	2.2	2.1	2.1	NS	0.003	NS
11	Handling body parts in connection with suicide or accidents	3.5	3.5	3.8	3.5	3.3	3.1	NS	NS	<0.001
12	Police intervention against crowd	4.8	4.6	4.5	3.5	3.3	3.0	<0.02	<0.001	NS
13	Working night shift	2.0	2.4	2.5	2.6	2.6	2.5	<0.001	<0.01	<0.002
14	That my colleague was suddenly shot, which led to serious injury or death	8.4	8.3	8.4	6.7	6.7	6.3	NS	<0.001	NS
15	To be injured by chemical substances	7.3	7.1	7.2	4.4	4.1	4.1	NS	<0.001	NS
16	Lacking a good supervisor	5.0	4.8	5.1	4.0	3.8	3.6	NS	<0.001	<0.01
17	That reorganization takes place	4.0	4.3	4.2	3.6	3.4	2.8	NS	<0.001	<0.001
18	That the division of roles is unclear in the unit or in the organization	4.2	4.1	4.1	3.5	3.4	3.0	NS	<0.001	<0.005
19	Living with guilt related to unsuccessful assignments	5.3	5.5	5.6	3.5	3.4	3.2	NS	<0.001	<0.001
20	Lacking advanced knowledge in police work	4.0	3.8	3.9	3.2	3.0	2.7	NS	<0.001	<0.005
21	That internal investigation is carried out when mistakes are made	4.9	4.9	4.9	3.5	3.4	3.1	NS	<0.001	<0.03
22	The cumbersome paperwork	3.6	3.5	3.3	3.8	3.5	2.8	NS	NS	<0.001
23	That supervisors do not do their job	4.3	4.4	4.4	3.5	3.4	3.1	NS	<0.001	<0.001
24	That the law is ineffective in dealing with interventions against offenders	2.9	2.7	2.8	4.6	4.3	3.6	NS	<0.001	<0.001
25	Having access to poor and outdated equipment	4.2	3.9	3.9	4.1	3.7	3.3	<0.01	NS	<0.007
26	To be away from family because of business travel within the country	2.3	2.4	2.4	2.3	2.3	2.3	NS	NS	NS
27	To be away from family because of business travel to another country	2.6	2.9	2.8	2.2	2.3	2.3	NS	<0.001	NS
28	To have a low degree of communication with one's life partner	4.8	4.9	5.0	3.9	3.8	3.5	NS	<0.001	<0.002
29	Not to receive support from one's life partner when it comes to conflicts at work	4.2	4.4	4.4	3.4	3.3	3.1	NS	<0.001	NS
30	Not sharing anything from my work in order to not disturb my family	2.6	2.6	2.8	2.4	2.5	2.2	<0.02	NS	NS
31	That family members have received death threats	6.7	6.7	6.9	6.4	6.3	6.2	NS	NS	NS
32	That the image of the police is distorted on the internet and in the media, such as TV and newspapers	3.2	2.9	3.5	3.2	3.3	2.7	<0.03	NS	<0.001
33	It is hopeless to be a police officer	1.4	1.5	1.6	2.1	2.1	1.6	<0.01	<0.001	<0.001
34	That I feel that police work has become much more difficult than before	2.2	2.3	2.4	2.7	2.6	2.1	NS	<0.001	<0.001
35	That the work culture within the organization disturbs me	2.6	2.5	2.5	2.6	2.4	2.0	NS	NS	<0.001
36	Performing new routines in connection with reorganization	2.6	2.7	2.7	2.6	2.4	2.1	NS	NS	<0.001
37	My colleagues’ view of me	2.3	2.4	2.5	2.1	2.2	2.1	NS	<0.001	NS
38	Regretting becoming a police officer	0.9	0.9	1.0	1.2	1.0	0.7	<0.03	<0.02	<0.001
39	To perform tasks that are against my own values	2.9	3.2	3.2	2.4	2.2	2.2	<0.03	<0.001	<0.01
40	Being harassed or receiving a death threat when off duty	4.2	4.5	5.1	3.8	4.8	4.8	<0.001	NS	NS
41	Not being able to do the job due to scarce personnel resources	4.6	4.8	4.7	4.9	4.6	4.1	NS	<0.02	<0.001
42	Meeting vulnerable people without being able to help them	4.7	4.7	4.8	4.2	3.8	3.5	NS	<0.001	<0.001

### 4.1. Interaction between time and country

We found an interaction between time and country for 23 items, suggesting that Sweden and Norway showed different patterns regarding changes in stress levels over time. These interactions can be classified into the following four patterns. There were 15 items that belonged to Pattern 1, 6 items to Pattern 2 and one item in each pattern 3 and 4.

#### Pattern 1: No difference over time in Norway and a decreasing trend in Sweden

**Figure 2:**
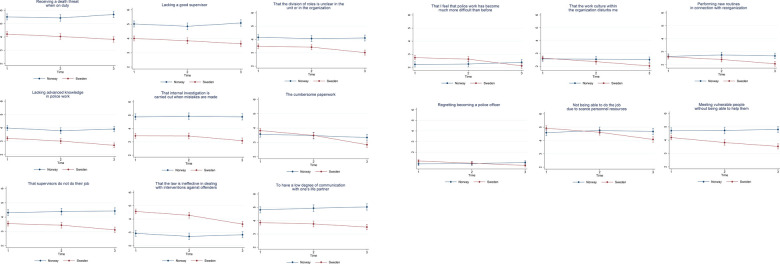
Estimated average marginal effects of stress items in pattern 1.

There were 15 items that showed no difference over time in Norway but decreased in Sweden. (Figure 2).

6. Receiving a death threat when on duty
16. Lacking a good supervisor
18. That the division of roles is unclear in the unit or in the organization
20. Lacking advanced knowledge in police work
21. That internal investigation is carried out when mistakes are made
22. The cumbersome paperwork
23. That supervisors do not do their job
24. That the law is ineffective in dealing with interventions against offenders
28. To have a low degree of communication with one's life partner
34. That I feel that police work has become much more difficult than before
35. That the work culture within the organization disturbs me
36. Performing new routines in connection with reorganization
38. Regretting becoming a police officer
41. Not being able to do the job due to scarce personnel resources
42. Meeting vulnerable people without being able to help them

#### Pattern 2: an increasing trend in Norway and a decreasing trend in Sweden

**Figure 3:**
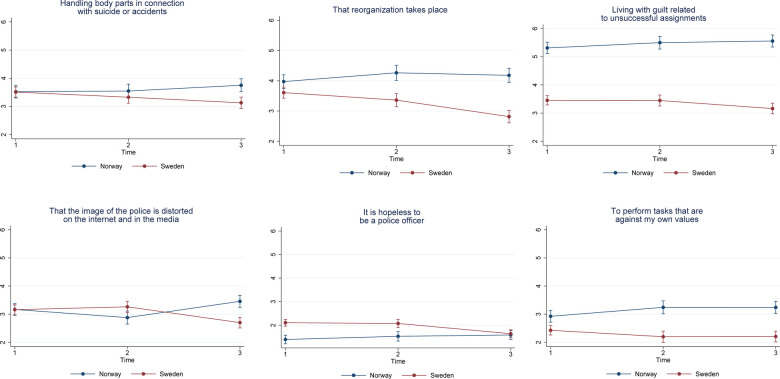
Estimated average marginal effects of stress items in pattern 2.

We found 6 items that in average increased over time in Norway but showed a decreasing trend over time in Sweden (Figure 3).

11. Handling body parts in connection with suicide or accidents
17. That reorganization takes place
19. Living with guilt related to unsuccessful assignments
32. That the image of the police is distorted on the internet and in the media, such as TV and newspapers
33. It is hopeless to be a police officer
39. To perform tasks that are against my own values

#### Pattern 3: decreasing trend for both Sweden and Norway

**Figure 4:**
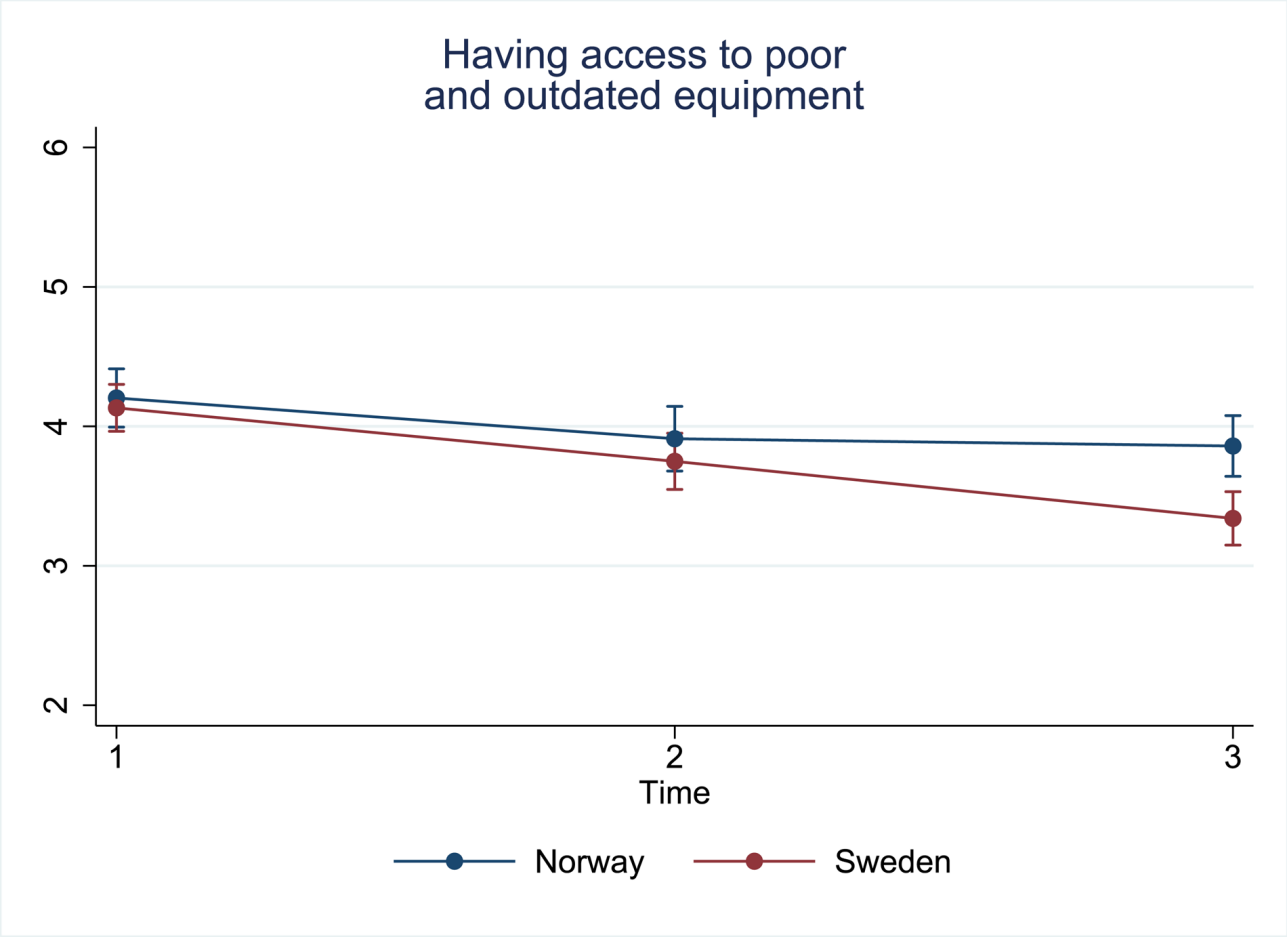
Estimated average marginal effects of stress items in pattern 3.

There was one item for which the mean value decreased over time in both countries (Figure 4).

25. Having access to poor and outdated equipment

#### Pattern 4: increasing trend in Norway and no change over time in Sweden

**Figure 5:**
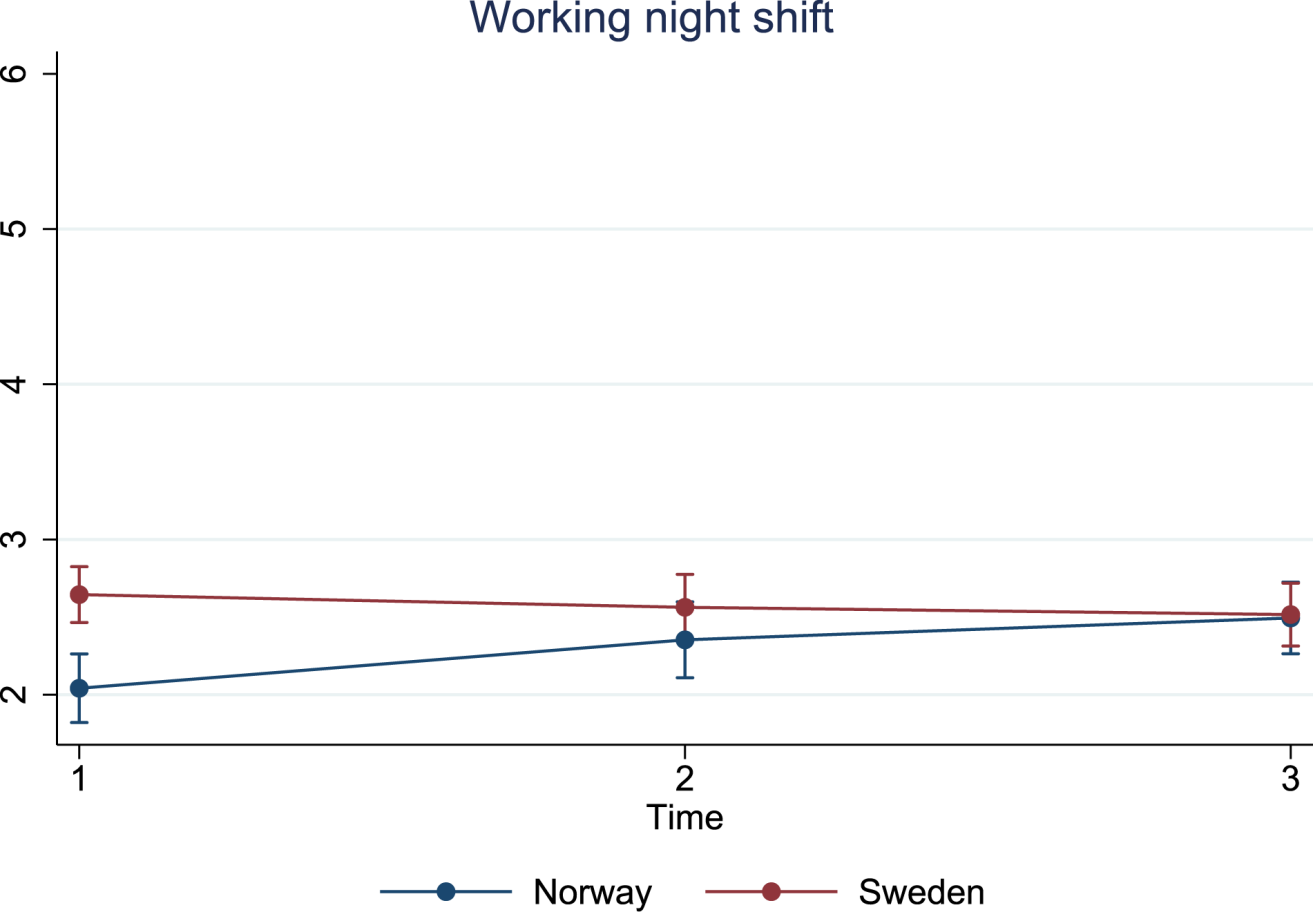
Estimated average marginal effects of stress items in pattern 4.

There was one item with an increasing trend over time in Norway but no change in Sweden (Figure 5).

13. Working night shift

## 5. Discussion

The aim of this study is to compare different types of stress among police officers in Norway and Sweden and investigate how the pattern of experiencing stress has changed over time in these countries. Both countries are highly developed and are economically comparable, but they hold significant disparities in terms of societal problems and population dynamics. Prior research tends to consider police work in northern countries in a similar manner. Researchers have also emphasized the extent to which the police in Nordic countries are renowned for emphasizing “soft policing” based on notions of prevention, social inclusion and an equal relationship between members of the civil society and the police (39). In general, Nordic police are endowed with a high level of public trust, and the general public perception is that discrimination and differential treatment rarely exist (13, 40, 41). We provided empirical evidence of potential differences in police work between the two countries that demand specific attention from their respective authorities. We found that Norwegian police officers experienced low stress (score of 1–3.9) for 21 items, such as “Regretting becoming a police officer” and “Working night shift”; medium stress (score of 4–6.9) for 19 items, such as “Having access to poor and outdated equipment “ and “Threats of physical violence”; and high stress (score of 7–9) for 2 items, namely, “To be injured by chemical substances” and “That my colleague was suddenly shot, which led to serious injury or death.” Contrary to Norway police, Swedish police had 32 items under the low stress category (e.g., “Regretting becoming a police officer”), 10 items in the medium level category (e.g., “Receiving a death threat when on duty”), and no item in the high level stress category. On average, Norwegian police officers reported higher levels of stress than their Swedish counterparts. Burke and Mikkelsen (42) reported that Norwegian police officers experience high levels of cynicism because of job stress and burnout. Findings from a nationwide survey of 3,272 Norwegian police personnel exploring the association between job stress and physical and mental health showed that frequent exposure to work stress is associated with health problems (43). The results showed that Norwegian police have high levels of musculoskeletal health problems, which are mainly associated with the frequency of job pressure and lack of support. The report concluded that daily routine work, as well as police operational duties, must be considered when assessing job stress and police health. There is a lack of national surveys among Swedish police regarding job stress, but a number of investigations have reported experienced job demand and police stress as a source of poor mental health (11, 23). Further investigation is needed to determine whether differences in aspects such as police organizational settings in the two countries are associated with higher levels of stress among Norwegian police officers.

Another explanation could be the fact that Norwegian police officers do not carry service weapons in their belts; they store their service weapons in the patrol car and equip them when necessary. This might explain why Norwegian police officers reported significantly higher stress on items 4–6, 12, and 14 (threat and violence on duty) compared with Swedish police officers when there are no significant differences for item 40 (threat of violence off duty). In other words, there is a difference in stress when the officers are on duty, with only Swedish police being armed, but there are no differences when they are off duty and unarmed. Meanwhile, there is no difference between the countries in terms of item 25, which is related to poor or outdated equipment. This needs to be investigated further as the issue of armed or unarmed police involves more perspectives than officers' perceptions of stress. Finally, the long-term effects of being armed on police officers need to be considered at individual and societal levels.

The findings of this study show a decreasing trend over time for most of the items among Swedish police officers. By contrast, the findings reveal no change or even increase over time for some items among the Norwegian participants. A possible explanation for this could be related to the Swedish police reorganization into one national authority in 2015. The first data collection was conducted three years after the reorganization, and the last data collection was conducted five years after the reorganization. During the first few years after the transformation into the national police authority, many supervisors had to apply for new positions, and during this time, many supervisor positions were temporary, resulting in accountability being at risk or unclear. In addition, many previously well-functioning units were reorganized, possibly causing distress among the personnel. The organization might have stabilized five years after the reorganization. Some studies have reviewed police reforms. For example, Mendel and colleagues (44) found relatively strong evidence for a range of risks involved in police mergers, such as the risk of loss of skills and competence, risk of disruption to employer–employee relationships, and risk of problems being exacerbated by inadequate planning and management. In addition, the Swedish Agency for Public Management (Statskontoret), which is responsible for evaluating police reorganization, found that the new organization lacks management and communication (45).

Another explanation could be that during the study period, Swedish police authorities initiated several interventions with the goal of improving safety and security in society. In 2017, the Swedish government announced a broad political consensus that Sweden's internal and external security must be strengthened through public security and institutional protection (46). Given this mission, there is a need to enhance crime prevention efforts by practical and political actors and capacity building for police units as one of the main stakeholders in fighting crime.

Our investigation has several limitations. The sample comprised only patrolling police officers which limits the generalizability of the findings. Items were selected from a range of possibilities that are relevant to patrolling police officers whose tasks are unique and somehow different from other police employees. Including desk police officers would require a different set of items that specifically concerns the target group. Being a police officer is a complex and diverse profession, and it is impossible to capture all possible stressors in police work in one questionnaire. As the first step, we decided to focus on patrolling police officers, who deal with certain types of stressors and move forward to a broader range of police stressors in future research.

In future studies, it would be relevant to use multiple methods to develop quantitative and qualitative data (such as participant observations) to explain the differences in various types of stress between the two countries. There are several other important future research directions that this study did not cover. For example, it would be interesting to investigate how subjective experiences of stress are connected to societal forces and the kinds of interactions that take place between the public and the police.

## Data Availability

The datasets presented in this article are not readily available because The data includes sensitive information and according the ethical approval we cannot share it with third party.
